# Monitoring Spawning Activity in a Southern California Marine Protected Area Using Molecular Identification of Fish Eggs

**DOI:** 10.1371/journal.pone.0134647

**Published:** 2015-08-26

**Authors:** Alice E. Harada, Elise A. Lindgren, Maiko C. Hermsmeier, Peter A. Rogowski, Eric Terrill, Ronald S. Burton

**Affiliations:** 1 Marine Biology Research Division, Scripps Institution of Oceanography, University of California San Diego, La Jolla, California, United States of America; 2 Coastal Observing Research and Development Center (CORDC), Scripps Institution of Oceanography, University of California San Diego, La Jolla, California, United States of America; University of California Santa Cruz, UNITED STATES

## Abstract

In order to protect the diverse ecosystems of coastal California, a series of marine protected areas (MPAs) have been established. The ability of these MPAs to preserve and potentially enhance marine resources can only be assessed if these habitats are monitored through time. This study establishes a baseline for monitoring the spawning activity of fish in the MPAs adjacent to Scripps Institution of Oceanography (La Jolla, CA, USA) by sampling fish eggs from the plankton. Using vertical plankton net tows, 266 collections were made from the Scripps Pier between 23 August 2012 and 28 August 2014; a total of 21,269 eggs were obtained. Eggs were identified using DNA barcoding: the COI or 16S rRNA gene was amplified from individual eggs and sequenced. All eggs that were successfully sequenced could be identified from a database of molecular barcodes of California fish species, resulting in species-level identification of 13,249 eggs. Additionally, a surface transport model of coastal circulation driven by current maps from high frequency radar was used to construct probability maps that estimate spawning locations that gave rise to the collected eggs. These maps indicated that currents usually come from the north but water parcels tend to be retained within the MPA; eggs sampled at the Scripps Pier have a high probability of having been spawned within the MPA. The surface transport model also suggests that although larvae have a high probability of being retained within the MPA, there is also significant spillover into nearby areas outside the MPA. This study provides an important baseline for addressing the extent to which spawning patterns of coastal California species may be affected by future changes in the ocean environment.

## Introduction

Marine Protected Areas (MPAs) are rapidly becoming a key tool for the management of living marine resources. Because there is a cost to excluding exploitation of resources within these reserves, it is important that the efficacy of MPAs be monitored to confirm that they are accomplishing their various conservation and resource enhancement goals. In this study, we establish a baseline for monitoring the spawning activity of fish species in the marine protected areas (MPAs) located adjacent to Scripps Institution of Oceanography. The area under study was established as an academic research area in 1929, prohibiting the take of invertebrates and marine aquatic plants [1]. In accordance with the Marine Life Protection Act (MLPA) passed in 1999, California’s MPAs were redesigned to better protect the diversity of the oceans. The newly designed MPAs went into effect in southern California in 2012, establishing, among others, the San Diego-Scripps Coastal State Marine Conservation Area (SMCA) and Matlahuayl State Marine Reserve (SMR), which include the sampling area of this study. The San Diego-Scripps SMCA prohibits the take of living marine resources, with the exception of coastal pelagic species (such as Pacific sardine, Northern anchovy, and jack mackerel) by hook-and-line. Adjacent is the Matlahuayl State Marine Reserve, which prohibits the take of all living marine resources [2].

Numerous fish population studies have previously been conducted off California [3–8], yet there are a limited number of studies concerning fish populations in the MPAs offshore of Scripps Institution of Oceanography (SIO). One of the few studies from this region, in which nearshore fish were surveyed by quarterly diver surveys and trawls, was conducted prior to the establishment of the no-take MPAs in San Diego [9]. More recently, Hastings *et al*. detailed all of the fish species in the La Jolla area based on the Marine Vertebrate Collection of SIO as well as diver surveys of the area [10]. Adult and juvenile fish surveys such as these may observe species missed in egg surveys, since fish egg studies are limited to species with pelagic eggs. However, while adult fish surveys provide insight into the variety of fish that inhabit an area, fish egg studies establish which species use the MPA and surrounding waters as a breeding ground by observing species that spawn in the region [11]. In this way, fish egg studies can complement diver surveys and trawls.

Fish eggs and larvae, or ichthyoplankton, are useful indicators of the spawning habits and composition of fish communities [12, 13]. Fish eggs have been regularly collected off California since 1949, when the California Cooperative Oceanic Fisheries Investigations (CalCOFI) began conducting quarterly cruises to collect plankton from the California Current [14]. CalCOFI ichthyoplankton samples are taken by bongo net tows on a grid of offshore stations. Formalin-preserved eggs are identified based on morphological characteristics such as egg shape, egg size, and number of oil droplets [15–18]. Morphological methods can be convenient for identifying species with distinct eggs and have been successful, for example, in monitoring Pacific sardine and Northern anchovy populations in the California Current [4]. However, many species are difficult to distinguish based solely on egg morphology [18, 19]. In a study of 288 fish species with pelagic eggs, 70% of eggs were between 0.5 and 1.5 mm in length, and most of these were spherical and contained one oil droplet [20]. This may result in unidentified or misidentified eggs, especially of species that are closely related.

Due to the limitations of morphological identifications, molecular methods of egg identification are becoming increasingly common. Species-specific primers and oligonucleotide probes have been designed in previous studies to allow for efficient identification of fish eggs and larvae [21, 22]. These techniques are limited to the identification of a set of predetermined species, however, and species without designated primers or probes will be missed in these analyses. When dealing with a diverse range of species, therefore, direct sequencing of a target gene from each egg followed by comparison to an appropriate DNA barcoding database may frequently be the most efficient approach. DNA barcoding can distinguish between closely related species and has been successfully implemented in the identification of fish [23–29].

In this study, DNA barcoding was used to identify fish eggs collected off the Scripps Pier, located at the junction of the San Diego-Scripps Coastal SMCA and the Matlahuayl SMR, from August 2012 through August 2014. Eggs were collected multiple times per week in order to observe changes in species composition and abundance correlated with time of year, season, and water temperature. Following the study, several collection dates were identified for their high diversity or notable species. Focusing on these dates, probability exposure maps were produced to show where the eggs were most likely to have been spawned based on the surface currents on the days prior to collection [30]. Most of these maps showed a high probability of local retention within the MPA, indicating its utilization as a spawning site for many of the fish species we identified. Furthermore, probability maps were used to estimate the dispersal of larvae for 20 days following certain collection dates. These maps suggest that, while a majority of larvae are retained within the MPA, there is also some spillover to nearby areas. By combining regular, quantifiable collection methods, accurate egg identifications, and predictive current modeling, this study provides a clear picture of spawning within the MPAs. Furthermore, it establishes a baseline measure of species composition and spawning seasons that can be compared to future studies.

## Methods

### Sampling location, technique, and frequency

Beginning in August 2012, plankton samples were taken from the end of the Scripps Pier, located at the junction of the San Diego-Scripps Coastal SMCA and the Matlahuayl State Marine Reserve. The San Diego-Scripps Coastal SMCA extends from just south of Scripps Pier (32° 51.964’ N latitude) northward to 32° 53.000’ N, while the Matlahuayl SMR extends from 32° 51.964’ N to 32° 51.067’ N. Samples were collected by lowering a 505-micron mesh, one meter-diameter plankton net from the Scripps Pier. The net was lowered until it reached the seafloor. It was then raised out of the water and lowered three more times for a total of four pulls screening a water volume of approximately 16 cubic meters (based an average water depth of 5m). The sample in the cod end was collected, and with the aid of a dissecting microscope (10X), fish eggs were removed. At this point, Northern anchovy (*Engraulis mordax*) and Pacific sardine (*Sardinops sagax*) could be identified morphologically. Anchovy are the only species in Southern California with oval-shaped eggs. Sardine eggs are very large (~1.9 mm) with a distinct outer envelope. The rest of the eggs were stored at 4°C in 95% ethanol for at least 12 hours prior to further processing.

### Ethics statement

The permit for collection of plankton (including fish eggs) from the MPAs (#4564) was issued by the California Department of Fish and Wildlife. Fish eggs were collected via plankton net tows using a 505-micron mesh net. They were placed in ethanol for preservation after collection. IACUC only requires approval if vertebrate eggs are maintained until hatching, so animal welfare protocols were not required. No collected eggs or larvae were near the free-feeding stage, and no endangered or protected species were involved in this work.

### Processing and storing eggs

After storing in ethanol overnight, individual eggs were rinsed with deionized water and crushed in fifteen microliters of buffer (two-thirds Qiagen AE Buffer, one-third water) with a clean pipette tip to release DNA [22]. No further DNA extraction or purification was needed. Samples were stored at -20°C prior to PCR.

### PCR and DNA purification

**Fig 1:**
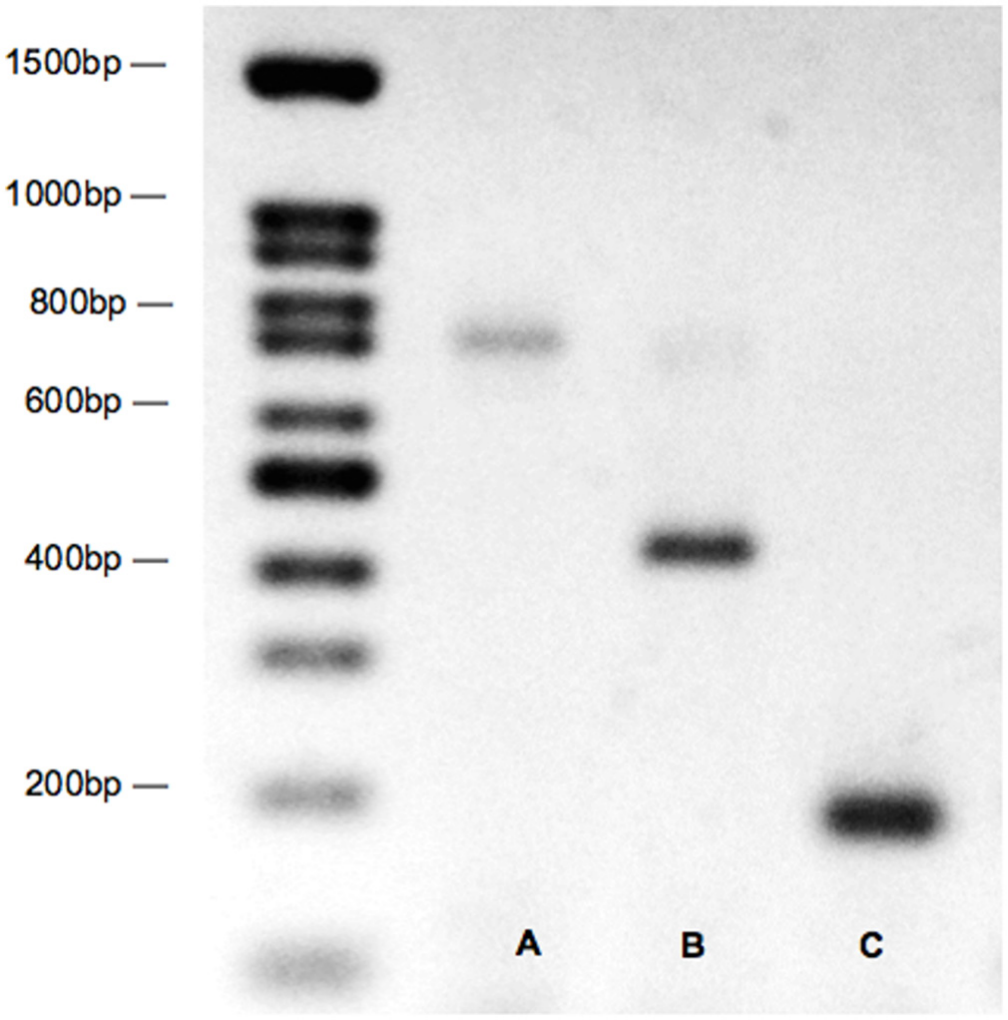
Gel electrophoresis image of fish egg PCR products using species-specific primers. Sample A is the amplified COI gene. Samples B and C identify *Citharichthys stigmaeus* and *Oxyjulis californica* eggs, respectively.

To amplify DNA, universal fish COI primers were used [29, 31]: COI VF1 forward primer (5′-TTCTCAACCAACCACAAAGACATTGG-3’) and COI VR1 reverse (5′-TAGACTTCTGGGTGGCCAAAGAATCA-3′). These primers produced an amplicon of 710 bp. Once the abundance of different species had been analyzed for several months, species-specific primers were designed to detect the most abundant species, speckled sanddab (*Citharichthys stigmaeus*) and señorita (*Oxyjulis californica*). These forward primers (*C*. *stigmaeus*: 5’-GCTCCCTCCCTCTTTTCTATTAC-3’; *O*. *californica*: 5’-GCCCCTGTTTGTCTGAGCTGTA-3’), used with the COI VR1 reverse primer, produced smaller amplicons (430 bp and 205 bp, respectively). The PCR reaction was carried out in a volume of 25 μl, including 12.5 μl of GoTaq Green Master Mix (Promega), the four primers at 5 pmol each, and one μl of DNA extract. The thermal cycler profile for the COI reaction was 95°C for 2 min, 35 cycles of 95°C for 30 s, 50°C for 45 s, and 72°C for 1 min, followed by 72°C for 5 min. Following PCR, samples were run on a 1.5% agarose gel and visualized with ethidium bromide or GelRed (Biotium) to detect presence of amplified DNA. After the species-specific primers were developed, smaller bands of appropriate length on the gel indicated that the sample was *C*. *stigmaeus* or *O*. *californica* (Fig 1).

Samples with only the 710 bp product were then purified using G-50 Fine Sephadex (GE Healthcare) spin columns, sequenced (out-sourced to commercial service), and identified using reference sequences published in GenBank (NCBI) or by the Barcode of Life Data System (http://www.barcodinglife.org). Supported by California Sea Grant (2004–2007), R. Burton and P. Hastings (Scripps Institution of Oceanography) obtained DNA barcodes from over 500 species of California marine fishes using specimens vouchered in the Scripps Institution of Oceanography Marine Vertebrates Collection; this work and follow-up efforts have resulted in nearly complete coverage of all but the most rarely observed species in California waters.

In order to assign a sequence to a species, we required that the sequence match a species in the database by at least 95% (if there were no other closely matched species) or 99% or higher if there were other species that were closely related. Closely related species differed at diagnostic nucleotide sites; for example, although *Citharichthys xanthostigma* and *C*. *sordidus* are closely related (99% identity at COI), alignments of multiple sequences from each species in the NCBI database reveals 5 species-diagnostic sites within the 660 bp barcode reference sequences. Although some of our sequences were not full length, coverage of two or more diagnostic nucleotides was considered adequate for accurately assigning these species. For a subset of samples that failed amplification with COI, the mitochondrial 16S ribosomal rRNA gene was amplified using forward primer 16Sar (5’-CGCCTGTTATCAAAAACAT-3’) and reverse primer 16Sbr (5’-CCGGTCTGAACTCAGATCACGT-3’) following the same PCR cycling conditions. The 16S amplicon was approximately 570 bp and was purified for sequencing as above.

### Data analysis

The total number of eggs collected each day was recorded along with species identifications for each collection. Information on water temperature was obtained from the Southern California Coastal Ocean Observation System (http://www.sccoos.org). To test the correlation of eggs collected and temperature, collections were separated into those above the mean temperature (17.8°C) and those below. We calculated the average number of eggs in each category and then performed a t-test to assess significance. The average eggs per season was also calculated and tested for significance using a one-way analysis of variance (ANOVA) with a post-hoc Tukey’s HSD.

Permutational multivariate analysis of variance (PERMANOVA) was used in PRIMER 6 (PRIMER-E Ltd.) to assess differences in species assemblages between months. This is a nonparametric test that allows significance testing of non-normal data by using permutations to compute p-values. The analyses were conducted using Bray-Curtis dissimilarities, with log-transformed (x+1) data in order to reduce the weight of highly abundant samples. Post hoc pairwise tests were performed to test differences among months. A nonmetric multidimensional scaling ordination (NMDS) plot was produced in PRIMER to visually compare differences between monthly species assemblages. Two full years were analyzed, from September 2012 to August 2014. August 2012 was not included in this analysis as it was under-sampled when compared to other months.

### A Plume Exposure Hindcast Model

To estimate the locations of the fish spawnings that yielded the eggs collected at the Pier, we assumed that eggs would typically hatch within three days of spawning (based on observations of eggs collected off the Pier and maintained in the lab at 18°C) and developed a hindcast model using archived high frequency (HF) radar data of surface currents in the region around the La Jolla MPAs. A Lagrangian forward-in-time (FIT) particle trajectory, representing parcels of water, is computed in the time domain:

$$x\left(t\right)=\;\int_{t_{0}}^{t}\left(u\left(t^{\prime }\right)+\;\varepsilon^{u}\right)dt^{\prime }+x\left(t_{0}\right)=\;\sum_{k}\left(u\left(t_{k}\right)+\;\varepsilon_{k}^{u}\right)\Delta t+x\left(t_{0}\right)$$ (1)

$$y\left(t\right)=\;\int_{t_{0}}^{t}\left(v\left(t^{\prime }\right)+\;\varepsilon^{v}\right)dt^{\prime }+y\left(t_{0}\right)=\;\sum_{k}\left(y\left(t_{k}\right)+\;\varepsilon_{k}^{y}\right)\Delta t+y\left(t_{0}\right)$$ (2)

where *x(t) = [x(t)y(t)]*† and *u(t) = [u(t)v(t)]*† denote the location of the particle (i.e., water tracer) and the surface currents at the tracer location at a given time (*t*), respectively. Here, *t*_o_ is the initial time of the simulation and † denotes the matrix transpose. ε^u^ and ε^v^ are the random variables with zero mean and rms of ε. The diffusion parameter (ε^u^ and ε^v^) represents unresolved velocities as the uncertainty in the HF radar measurements (ε = 5 cm s-1).

FIT particle tracking models are commonly used by the oceanographic community to research processes that influence the transport of developing fish larvae and eggs prior to settlement [32]. However, these models are computationally expensive for identifying the source of particles arriving at a fixed location. A more efficient option is to track particles backwards-in-time (BIT) from their destination to their source. A BIT trajectory model can be computed in the time domain as:

$$x\left(t\right)=\;\int_{t_{0}}^{t}\left(u\left(t^{\prime }\right)+\;\varepsilon^{u}\right)dt^{\prime }+x\left(t_{0}\right)=\;\sum_{k}\left(u\left(t_{k}\right)+\;\varepsilon_{k}^{u}\right)\Delta t+x\left(t_{0}\right)$$ (3)

$$y\left(t\right)=\;\int_{t_{0}}^{t}\left(v\left(t^{\prime }\right)+\;\varepsilon^{v}\right)dt^{\prime }+y\left(t_{0}\right)=\;\sum_{k}\left(v\left(t_{k}\right)+\;\varepsilon_{k}^{y}\right)\Delta t+y\left(t_{0}\right)$$ (4)

where *t*_0_ is the initial time of the simulation and *t* now represents the backward time step.

HF radar-observed ocean surface currents were used to drive the transport model [33]. The uncertainty of the estimated coastal current field is approximately 8.6 cm s^-1^, which is consistent with reported root-mean-square (rms) errors between surface current measurements derived from HF radars and drifter velocity observations [34–36].

Previous work has used the forward-in-time version of this surface transport model to assess the fate and transport of several discharges in the San Diego/Tijuana border region during high fecal indicator bacteria (FIB) events from April 2003 to March 2007. The model’s skill in assessing water quality in the surf zone was evaluated using receiver operating characteristic (ROC) analysis, which showed 70% accuracy over a four-year period [36]. Additionally, Rogowski *et al*. utilized the surface transport model to determine potential stormwater exposure areas within the boundaries of MPAs [37]. High probability exposure areas were delineated by estimating the probabilistic spatial extent of 20 river discharges along the southern California coast.

The Monte Carlo simulations using the formulations in Eqs 3 and 4 were computed using 50 water tracers constantly being released each hour at the source location. To transport the numerical parcels of water near the coastal boundary we use an along-coast projection of currents inshore of the 1 km boundary, which is the nearshore extent of the HF radar’s observations (Eq 5). The coastal exposure kernel (CEK, P) defined as the relative probability of plume exposure computed from the ratio of the number of water tracers at a given location *F(x*,*y)* to the total number released at the source location, expressed in percent is [36]:

$$P\left(x,y\right)=\frac{F\left(x,y\right)}{\text{max}\left[F\left(x,y\right)\right]}\;\times 100$$ (5)

## Results

### Species composition

Overall, 21,269 eggs were collected in 266 collections in the period from August 23, 2012 to August 28, 2014. Of those, 13,249 were successfully sequenced and 39 fish species were identified. Table 1 lists all of the species found in the survey ordered by abundance. The most commonly found species were the speckled sanddab (*Citharichthys stigmaeus*), señorita (*Oxyjulis californica*), Pacific sardine (*Sardinops sagax*), and Northern anchovy (*Engraulis mordax*). Many of the remaining species were flatfish (e.g., *Citharichthys* spp., *Paralichthys californicus*) that commonly live on the soft substrates characteristic of our sampling location. Others are typically found near kelp forests (e.g., *Paralabrax clathratus*, *Girella nigricans*), such as the La Jolla Kelp Forest immediately south of our sampling site. We did not see species that are commonly found offshore, such as yellowtail *(Seriola lalandi)*, consistent with the idea that most of the eggs we find are spawned close to shore.

**Table 1:** Complete species list. List of all species collected (in order of egg abundance), total number of eggs collected, and number of collections (of 266 total) in which eggs were found.

Species	Common name	Number of eggs collected	Number of collections
*Citharichthys stigmaeus*	Speckled Sanddab	4317	228
*Oxyjulis californica*	Señorita	4075	134
*Sardinops sagax*	Pacific Sardine	1249	31
*Engraulis mordax*	Northern Anchovy	639	40
*Xenistius californiensis*	Californian Salema	541	40
*Menticirrhus undulatus*	California Corbina	394	52
*Citharichthys sordidus*	Pacific Sanddab	393	106
*Roncador stearnsii*	Spotfin Croaker	258	43
*Paralichthys californicus*	California Halibut	234	89
*Halichoeres semicinctus*	Rock Wrasse	230	64
*Seriphus politus*	Queenfish	210	32
*Trachurus symmetricus*	Pacific Jack Mackerel	112	20
*Hypsopsetta guttulata*	Diamond Turbot	85	54
*Paralabrax clathratus*	Kelp Bass	82	36
*Semicossyphus pulcher*	Sheephead	80	39
*Citharichthys xanthostigma*	Longfin Sanddab	57	32
*Cheilotrema saturnum*	Black Croaker	40	22
*Scomber japonicus*	Chub Mackerel	38	13
*Genyonemus lineatus*	White Croaker	33	12
*Anisotremus davidsonii*	Xantic Sargo	25	9
*Peprilus simillimus*	Pacific Pompano	24	16
*Cynoscion parvipinnis*	Shortfin Corvina	23	12
*Chilara taylori*	Spotted Cusk-eel	17	14
*Atractoscion nobilis*	White Seabass	16	7
*Symphurus atricaudus*	California Tonguefish	15	10
*Paralabrax nebulifer*	Barred Sand Bass	12	10
*Xystreurys liolepis*	Fantail Sole	11	3
*Umbrina roncador*	Yellowfin Croaker	10	5
*Pleuronichthys verticalis*	Hornyhead Turbot	6	4
*Pleuronichthys coenosus*	C-O Sole	5	5
*Caulolatilus princeps*	Ocean Whitefish	5	4
*Girella nigricans*	Opaleye	4	3
*Sphyraena argentea*	Pacific Barracuda	4	2
*Synodus lucioceps*	California Lizardfish	2	1
*Hypsoblennius jenkinsi*	Mussel Blenny	2	2
*Paralabrax maculatofasciatus*	Spotted Sand Bass	2	2
*Stereolepis gigas*	Giant Sea Bass	1	1
*Citharichthys fragilis*	Gulf Sanddab	1	1
*Hermosilla azurea*	Zebra-perch Sea Chub	1	1

### Annual and seasonal spawning trends

**Fig 2:**
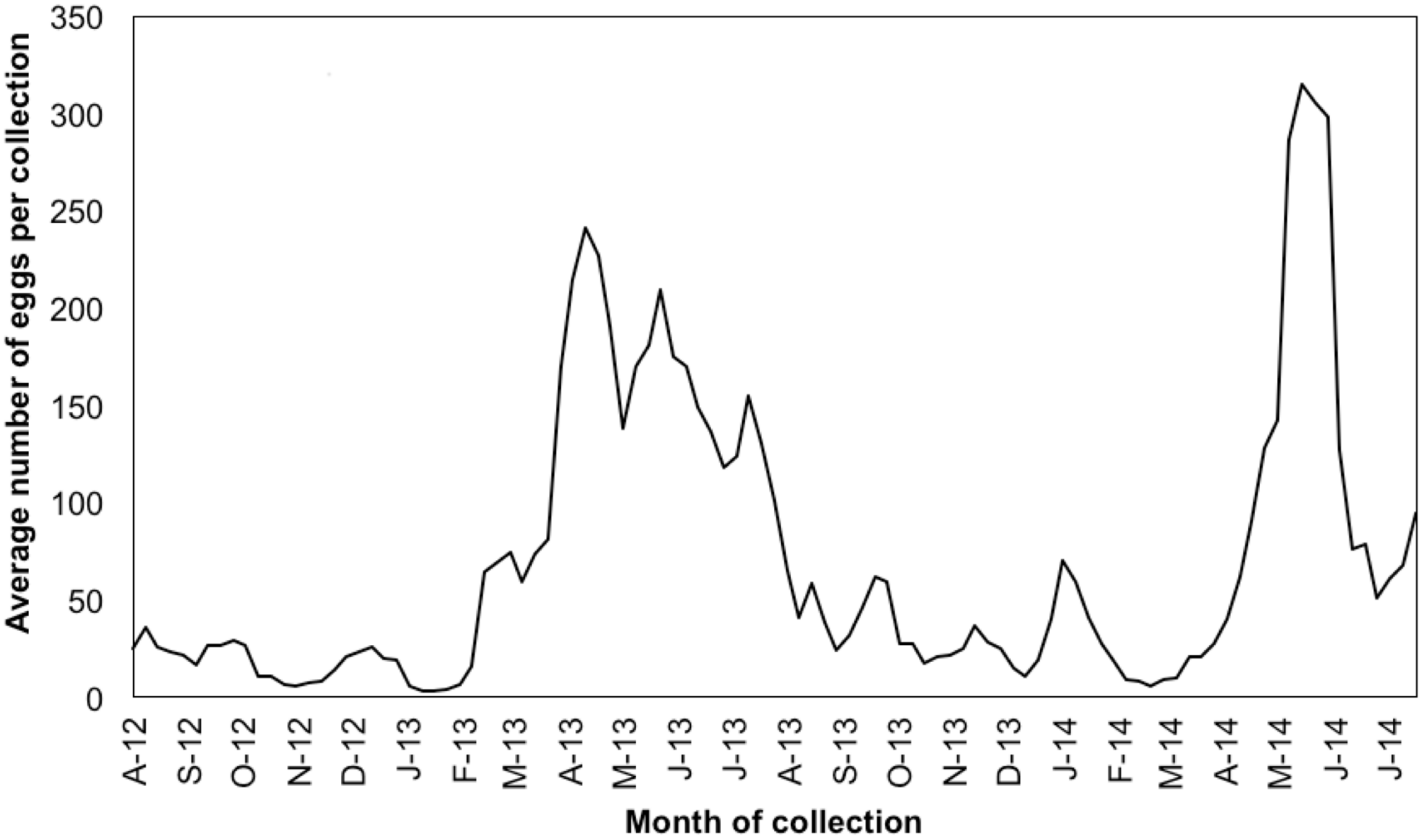
Graph of the average number of eggs per collection throughout the collection period from August 2012 to August 2014. This graph depicts a sliding window of the average eggs per collection in a three-week period overlapping by one week.

**Fig 3:**
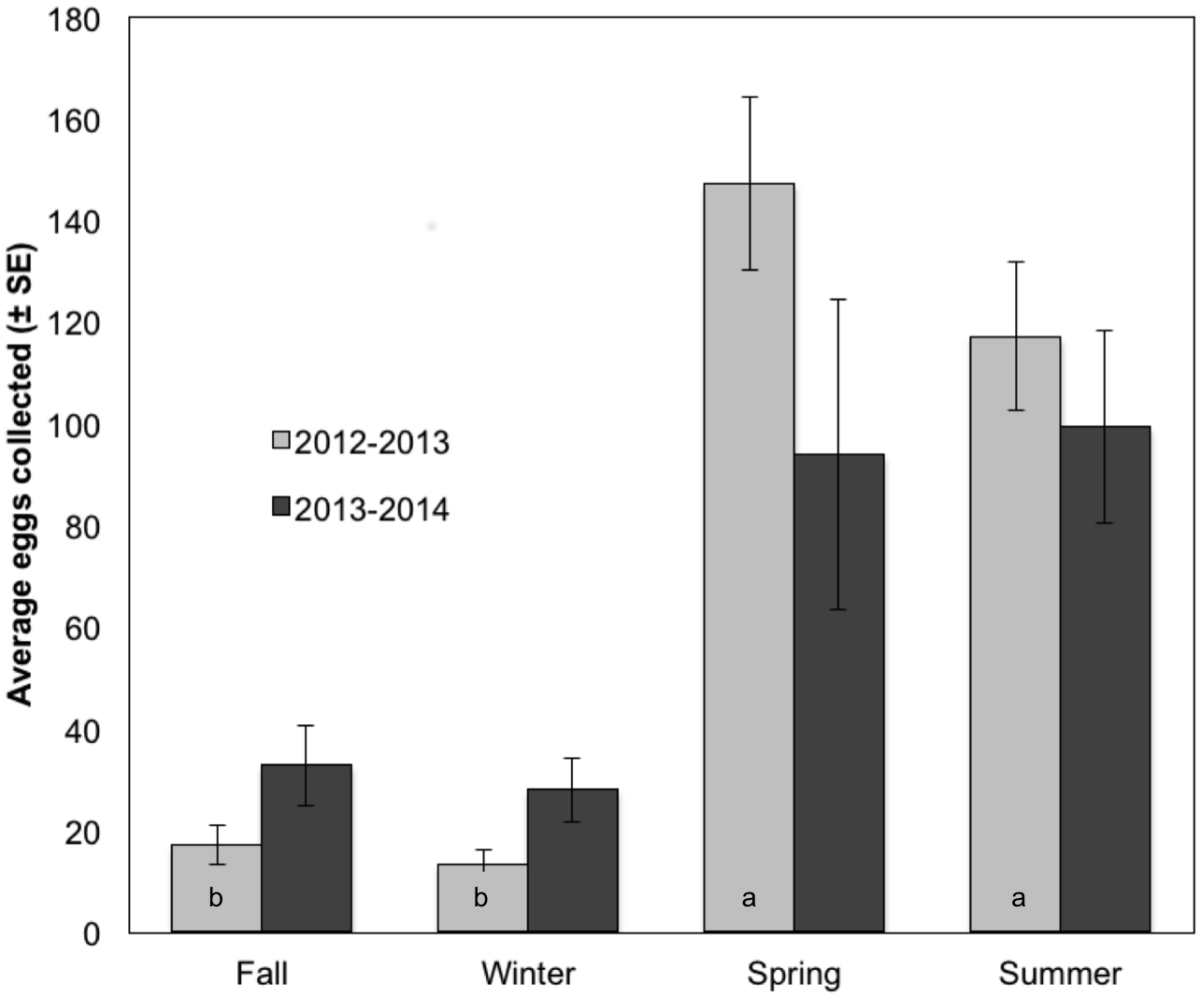
Graph of the average eggs collected (±SE) in each season of collection in the 2012–2013 collection year versus 2013–2014. In the first collection year, a one-way analysis of variance and post-hoc Tukey’s HSD revealed that there were significantly more eggs collected on average in spring and summer than in fall or winter (F_7,249_ = 10.74; spring vs. fall and winter, summer vs. winter, p < 0.0001; summer vs. fall, p < 0.001). There were no significant relationships in the second collection year.

Comparison of the 2012–2013 collection year to 2013–2014 reveals many similarities. Both years saw low average numbers of eggs collected in fall and winter and high spawning in spring and summer (Figs 2 and 3). Table in S1 Table lists all species with 10 or more eggs in the total survey arranged by month and shaded according to the proportion of collections they appeared in during a given month to reveal peak spawning seasons for each species. A few species spawn year-round, including the speckled sanddab *(C*. *stigmaeus)*, Pacific sanddab *(Citharichthys sordidus)*, California halibut *(Paralicthys californicus)*, diamond turbot *(Hypsopsetta guttulata)*, and longfin sanddab *(Citharichthys xanthostigma)*. However, most species spawn in a period that appears to be strictly defined. For example, although we only found 10 eggs of yellowfin croaker *(Umbrina roncador)*, the highest number of yellowfin croaker eggs in each year was found on June 19. Remarkably, in both years we observed the highest diversity of species in the June 19 collections.

**Fig 4:**
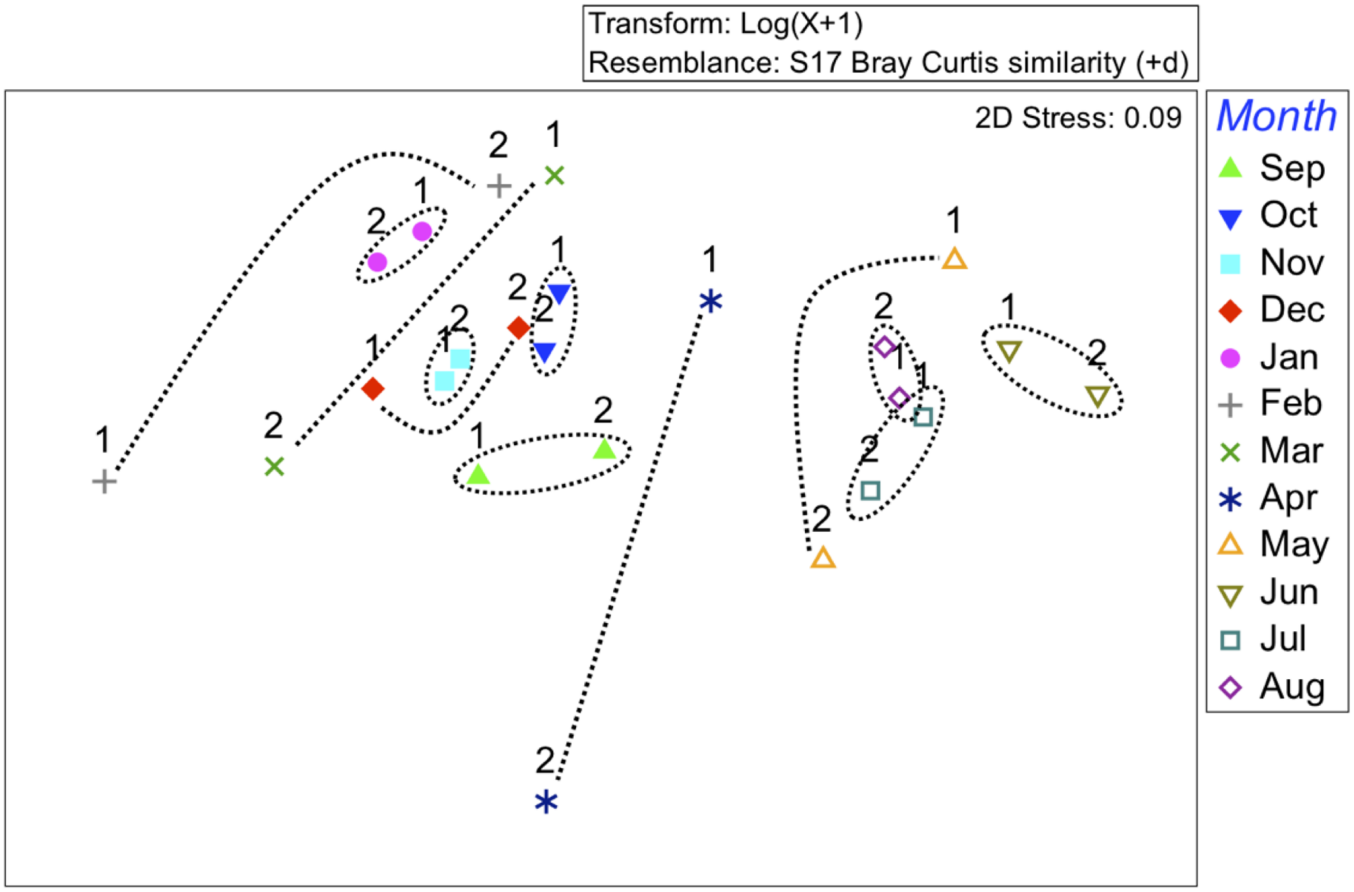
Nonmetric Multidimensional Scaling ordination (NMDS) plotted from average abundances of fish eggs for each species per month. Based on Bray-Curtis dissimilarities from log-transformed (x+1) data. The stress value indicates that the plot gives an adequate representation of the data. Numbers “1” or “2” above each point represent the first or second sampling year. Dotted lines connect or encircle the same months.

A multivariate analysis of community structure averaged by months over the two years of sampling reveals clustering of months between years (Fig 4). Although the communities vary dramatically across seasons, June 2013 and June 2014 (for example) are nearest neighbors in the MDS plot and are clearly distinct from even July and August samples. This tight clustering between the two sampling years is observed for each of the months of June through January, but breaks down in late winter and spring. This is consistent with the transitional and more variable nature of both the amount and species of eggs collected in spring months compared to the remainder of the year. For example, in the first year of sampling we saw Northern anchovy (*E*. *mordax*) spawning from January through March, while in the second year anchovy spawning concluded in February. Using 999 permutations, PERMANOVA showed a significant relationship between months (PERMANOVA, pseudo-F = 3.49, p = 0.001) but not between the two sampling years (pseudo-F = 0.24657, p = 0.957). Pairwise Monte Carlo tests showed differences in the species assemblages between fall/winter and summer months (p < 0.05). This is supported by the NMDS plot, which shows clustering of summer months and fall/winter months with spring falling between the two clusters.

### Effect of temperature

**Fig 5:**
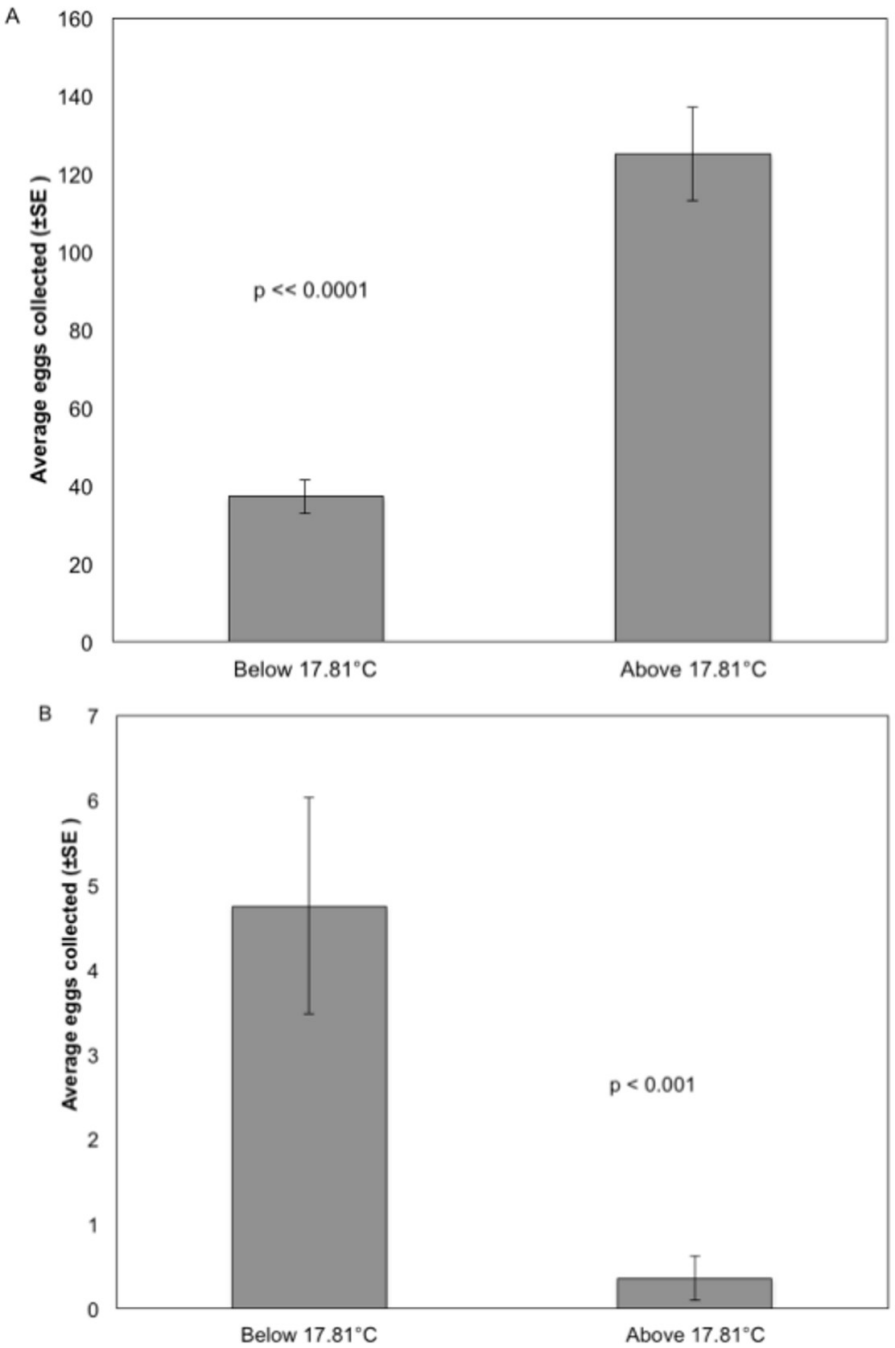
Bar graph of the average number of eggs (±SE) collected below and above the mean seawater temperature during the collection period. Graph A shows the average eggs collected from all species except Northern anchovy *(Engraulis mordax)*. There were significantly more eggs collected on average when the water temperature was higher than the mean (unpaired t-test, t_247_ = 6.919, p = 3.887e-11). Graph B shows the average anchovy eggs collected. There were significantly more anchovy eggs collected on average when the water temperature was below the mean (unpaired t-test, t_247_ = -3.4103, p < 0.001). Below average temperature n = 123 collections; above n = 126.

Seawater temperature is measured continuously from the Scripps Pier at a depth of ~2 m and digitally recorded approximately every 2 minutes. To find the temperature for each collection we took the average of the ten temperatures before and ten temperatures after the recorded collection time. The mean temperature across all collections was 17.8°C. We then determined the average number of eggs per collection when water temperature was either above or below this mean (Fig 5). Because anchovy are known to spawn in colder waters [38], we calculated the relationship of temperature and Northern anchovy *(E*. *mordax)* separately from the other species. Using an unpaired t-test, we found significantly more total eggs in collections taken when temperatures were above the mean temperature (t_247_ = 6.919, p = 3.89e-11); in contrast, significantly fewer anchovy eggs were collected when temperatures were above the mean (t_247_ = -3.4103, p = 0.00076). The average seawater temperature on days that anchovy eggs were collected was 14.7°C.

### Egg dispersal estimates utilizing a surface transport model

**Fig 6:**
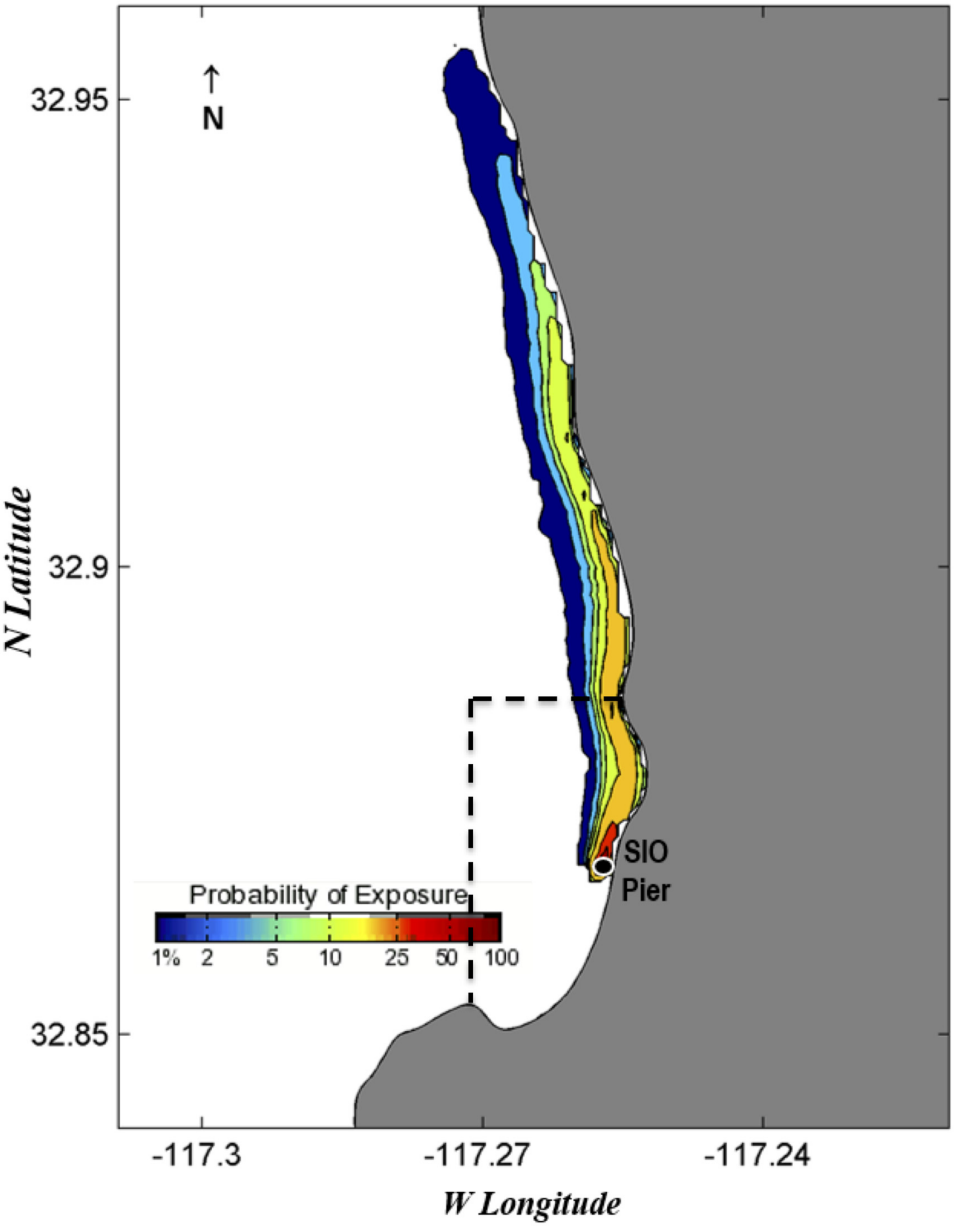
Probability map of predicted fish egg dispersal prior to collection: June 16–19, 2013. Dotted lines depict the boundaries of the MPAs: the San Diego-Scripps SMCA to the north and the Matlahuayl SMR to the south.

**Fig 7:**
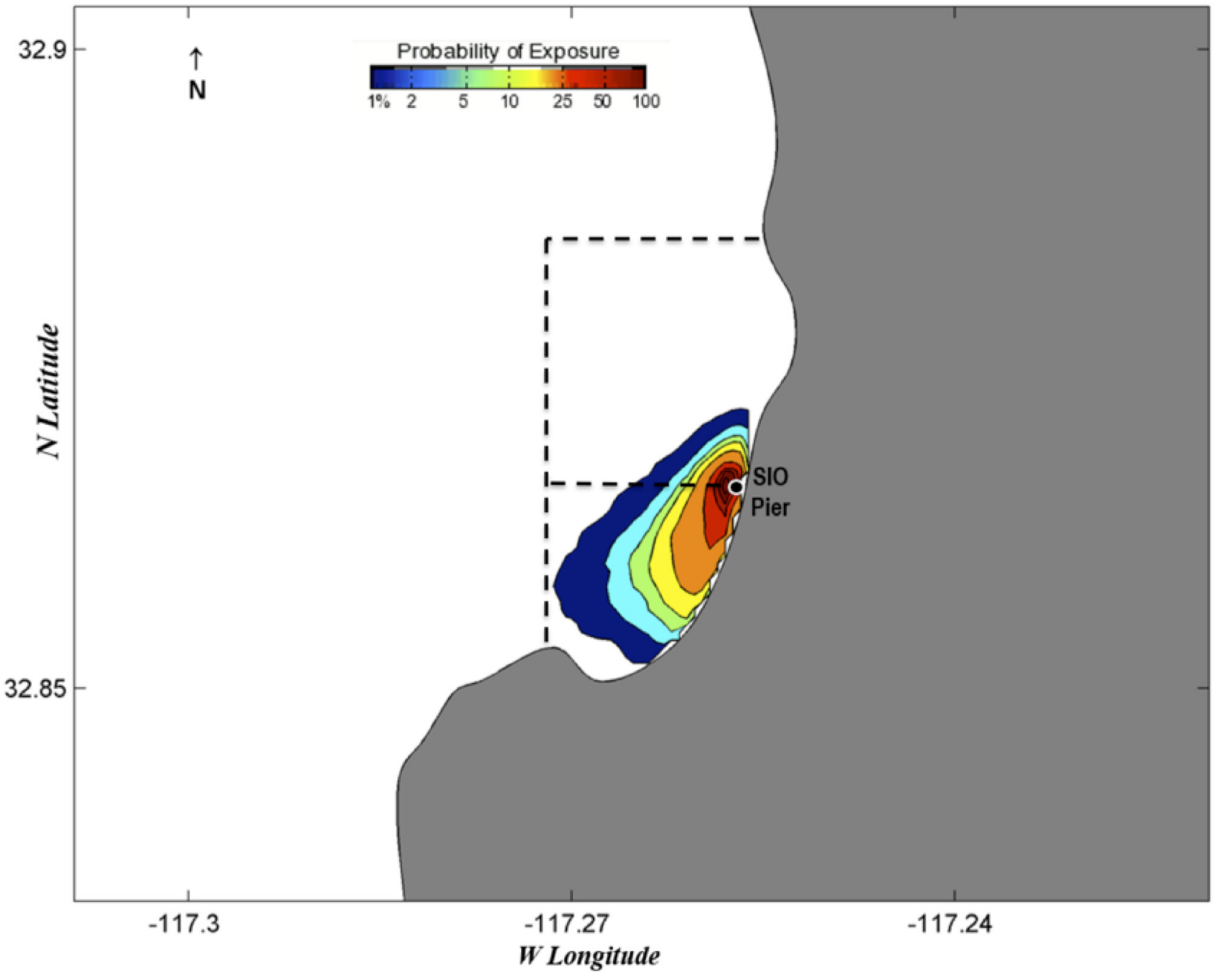
Probability map of predicted fish egg dispersal prior to collection: August 18–21, 2013. Dotted lines depict the boundaries of the MPAs: the San Diego-Scripps SMCA to the north and the Matlahuayl SMR to the south.

**Fig 8:**
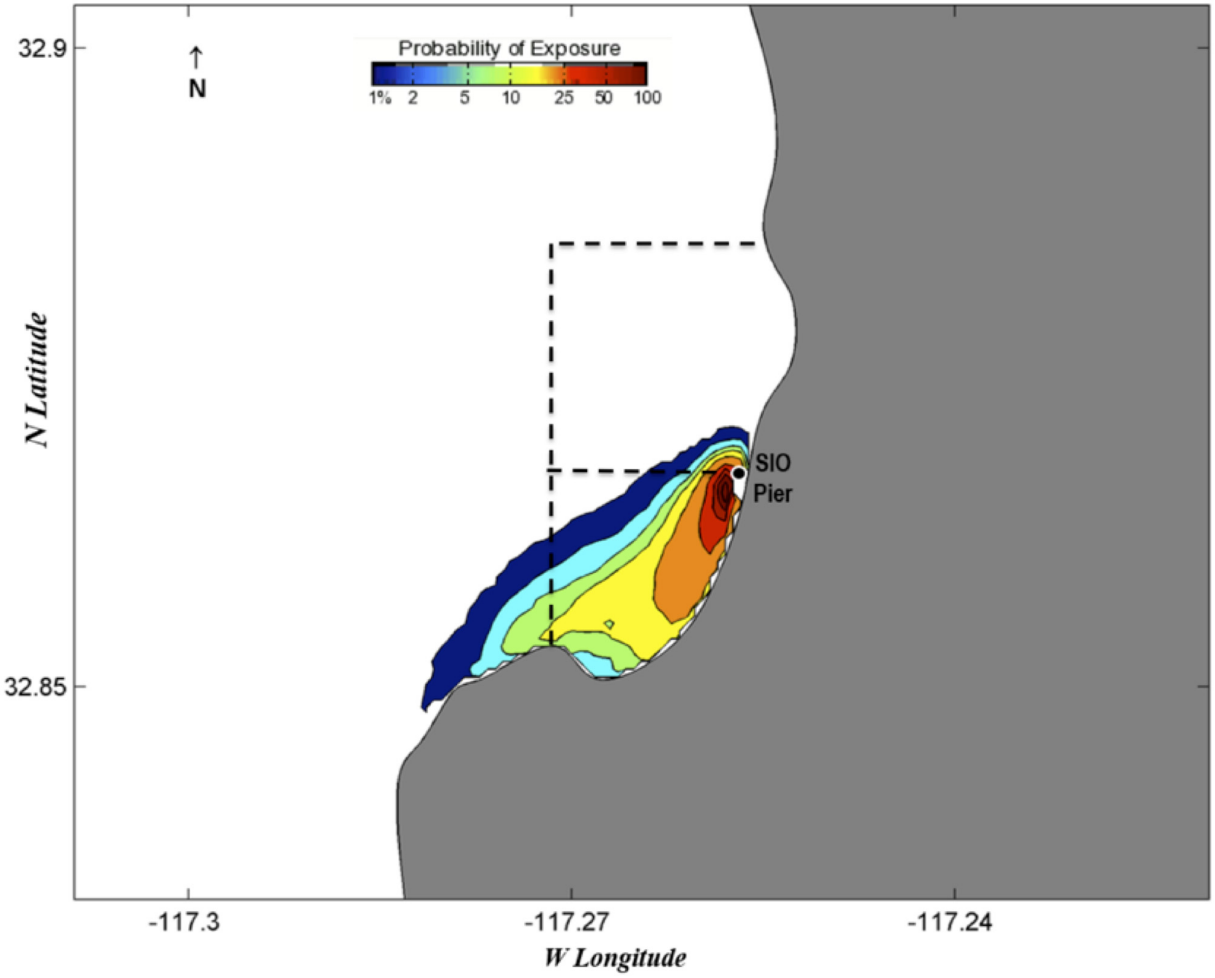
Probability map of predicted fish egg dispersal prior to collection: August 31-September 3, 2013. Dotted lines depict the boundaries of the MPAs: the San Diego-Scripps SMCA to the north and the Matlahuayl SMR to the south.

**Fig 9:**
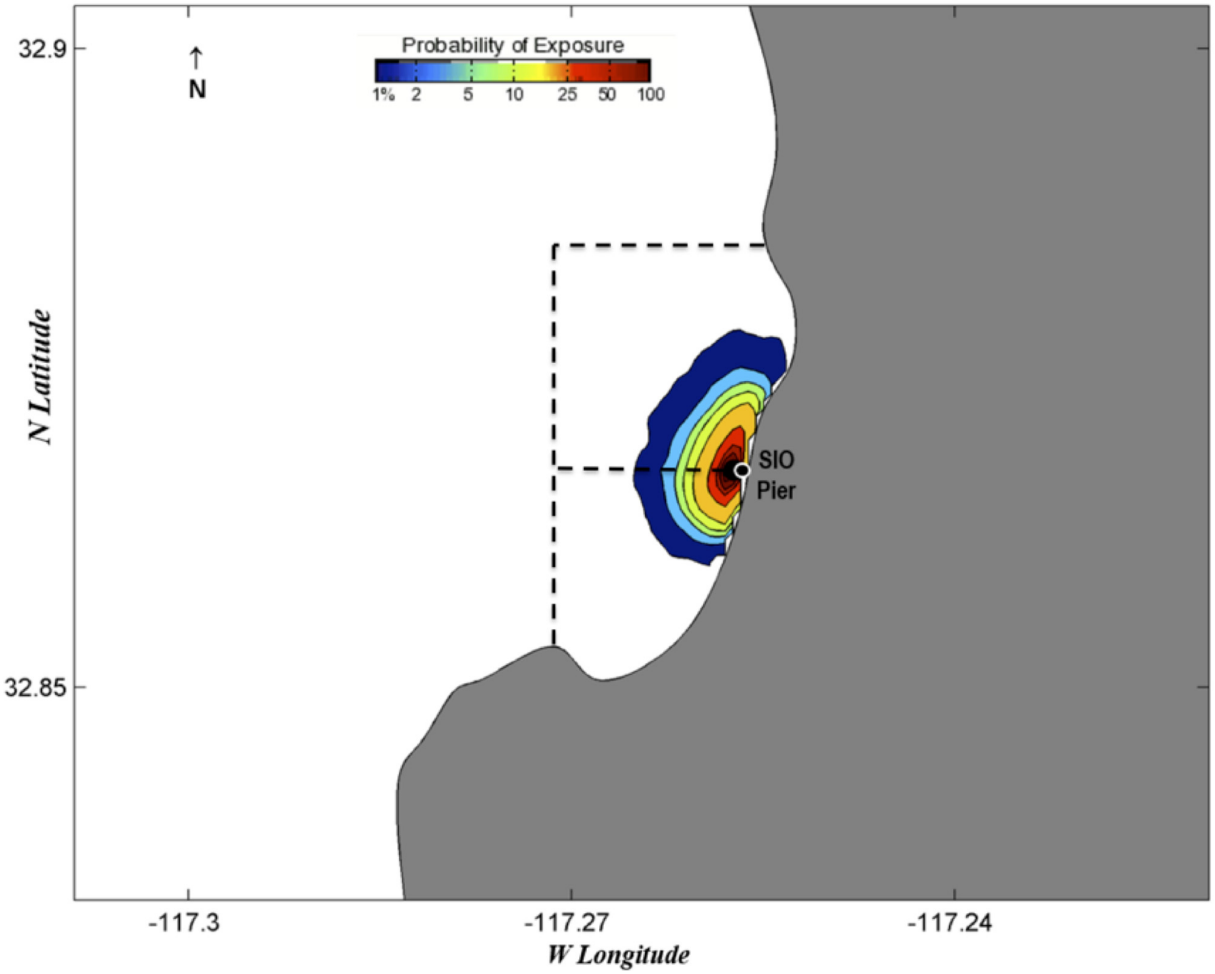
Probability map of predicted fish egg dispersal prior to collection: February 1–4, 2014. Dotted lines depict the boundaries of the MPAs: the San Diego-Scripps SMCA to the north and the Matlahuayl SMR to the south.

**Fig 10:**
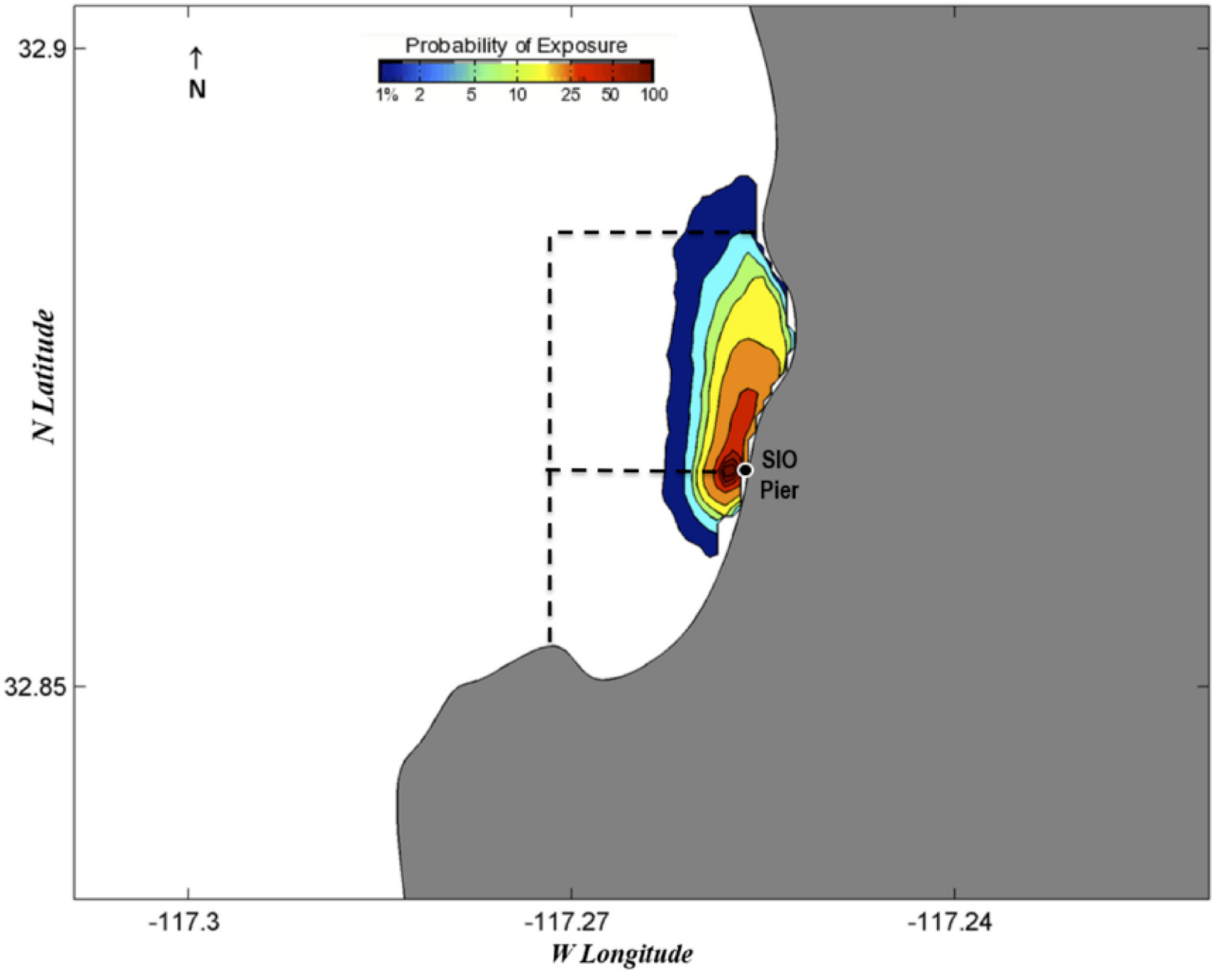
Probability map of predicted fish egg dispersal prior to collection: June 16–19, 2014. Dotted lines depict the boundaries of the MPAs: the San Diego-Scripps SMCA to the north and the Matlahuayl SMR to the south.

Probability exposure maps estimate the probability of eggs being spawned in certain locations three days prior to chosen collection dates (Figs 6–10). Given the species collected and the ambient water temperatures, three days approximates the hatching time of most eggs we collect [39]. We chose collection dates due to their high species diversity (June 19, 2013; February 4, 2014; and June 19, 2014) or the presence of kelp forest species (August 21, 2013, and September 3, 2013). Of those dates, all but June 19, 2013, showed with high likelihood that all eggs would have been spawned within or almost completely within the MPA boundaries. June 19, 2013, showed a high probability of eggs being spawned within the MPA but also an effect of a southward current that could have brought eggs into the MPA from the north (Fig 6). August 21, 2013 (Fig 7), and September 3, 2013 (Fig 8), showed influence from northward currents, however we note that small-scale current variability (< 1 km) near La Jolla Point would increase estimation errors in the along-coast current velocity projections due to the lack of nearshore observations by the HF radar [40]. On those days we detected kelp-associated species in our pier sampling, suggesting eggs flowing from the south could be coming from a kelp forest just south of our sampling site. It may be the case that the nearby kelp forest plays a part in slowing down currents within the cove, contributing to retention of eggs [41].

**Fig 11:**
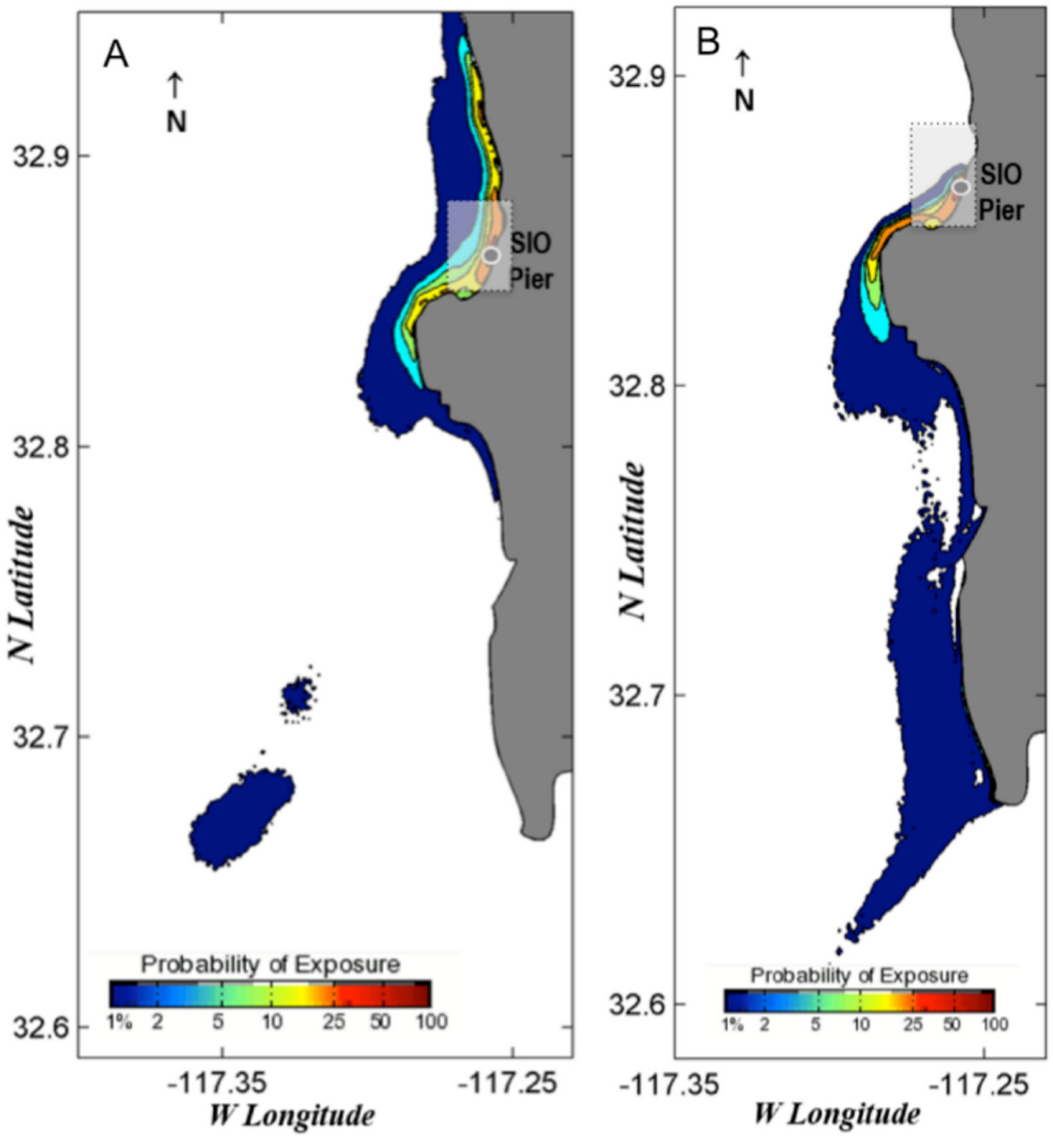
Probability maps of predicted larval fish dispersal. Both maps show the results of the predictive model run for twenty days following a collection date to visualize the probable distribution of larvae after hatching. The white box denotes the approximate boundaries of the MPAs. Dates are (A) June 19-July 9, 2013 and (B) February 4–25, 2014.

Additionally, two probability maps utilizing the forward-in-time surface transport model (Eqs 1 and 2) were generated with a 20-day event window (statistical distribution based on 20-days of estimated particle trajectories after pier collection date) to estimate pre-settlement spatial dispersion of larvae after hatching. We selected collections on June 19, 2013, and February 4, 2014, for their high diversity and because they represented two different collection years and seasons. Given the model’s nearshore limitation, we use the maps to illustrate a conservative estimate of exposure, suggesting that there is both a high probability of larval retention within the MPAs as well as spillover to nearby areas (Fig 11).

### Re-sequencing using 16S rRNA primers

Nearly all sequencing was done with the cytochrome oxidase subunit one (COI) universal primers. A potential bias could be introduced into the study if these primers fail to amplify specific species that occur in the collections. This is somewhat unlikely since these same primers were used in the sequencing of vouchers that are in the barcode database. However, to ensure that there was no amplification bias with COI, 1,066 samples from various collections throughout the sampling period that failed to amplify with COI were re-sequenced using 16S rRNA universal primers. Of these, 165 samples still did not amplify, suggesting that at least 15% of the failed PCRs were likely due to poor template quality (e.g., failed DNA extraction, degraded DNA, or PCR inhibitors present). We then compared the composition of species in the re-sequenced samples to the composition of species initially sequenced with COI. If we assume that failure with COI was random (i.e., not biased by species), we would predict that the distribution of species identified in the 16S sample would be similar to that of the COI sample. We obtained 16S sequences from 27 species, and as with COI, *C*. *stigmaeus* was dominant (34.7% of the 16S sequences compared to 32.8% of the COI sequences, X^2^, = 2.04, 1 df, p > 0.1). Statistically, the rank order of species in the two distributions did not differ significantly (Wilcoxon signed rank test, n = 27, T = 110, p > 0.05). The only notable evidence of bias concerned a single species, the hornyhead turbot *(Pleuronichthys verticalis)*, which was only found with 16S sequencing (6 of 901 eggs sequenced for 16S versus 0 of 12,348 sequenced for COI, X^2^ with Yates correction = 68.2, 1 df, p < 0.001).

## Discussion

In this study, we used DNA barcoding to identify fish eggs collected off the Scripps Pier over two years of approximately twice-weekly sampling. This time series reveals several spawning trends. Seasonal changes had a profound impact on spawning, with a significantly higher average number of eggs found in spring and summer than in winter or fall. In general, we found noteworthy consistency between the two years of the study, with many species spawning on nearly the same date in both years. Remarkably, the dates with the greatest species diversity in each year were both June 19. These consistencies between the two sampling years, along with the data from the NMDS plot and PERMANOVA, suggest that seasonality is a key driver of the spawning of these species.

In addition to seasonal influence, we also found a significant effect when examining the correlation between temperature and the number of eggs collected. There were significantly more eggs spawned (all species except anchovy) when seawater temperature was above average than when it was below. It is difficult to determine whether season or temperature has a greater affect on spawning since they are correlated; a longer time series including years with anomalous seasonal temperature may be able to resolve the relative importance of temperature versus season.

Recent work on the analysis of marine microbial communities has increasingly employed high throughput (“next-generation” sequencing or NGS) approaches. For a variety of reasons, Sanger sequencing best served our purposes. Our goal was to quantitatively assess the number of eggs and their species-level diversity in each of our 266 collections. If egg samples are pooled prior to DNA extraction (as done in NGS approaches), the quantitative analysis of the relative number of eggs of each species could be compromised by small differences in the efficiency of PCR leading to over- or under-representation of different species in the amplicon library [42]. Even with no PCR bias, different ages of eggs will have different cell numbers such that one 3-day old egg could well contribute 10-100X more mtDNA copies to the sample than several eggs that are only hours old, again distorting the estimates of egg numbers (in favor of egg biomass). Finally, many or our samples were heavily dominated by a single (or few) species yet contained a significant number of species represented by a single egg; for example, of the 15 species observed in the June 19, 2013, collection, 7 species were identified from only a single egg. Although NGS sequencing of amplicons may reveal the full extent of diversity, the required sequencing coverage and cost required to observe singleton eggs for each sample date is not clear. Since even a single egg reveals a spawning event, singletons are quite important to our goal of determining which species are spawning in the vicinity of the La Jolla MPAs.

Another important point to note in this study is the fairly large number of eggs collected that were not sequenced (approximately 38%). Though this can be attributed to several errors along the path from collection to sequencing, the highest portion of these came from amplification failures during PCR. To ensure that this was not due to our universal primers selectively amplifying some species and not others, we amplified and sequenced samples from a variety of collections throughout the sampling period using 16S rRNA primers. 16S is a commonly used gene for universal amplification but not the main gene sequenced in this study because it is less widely established for fish barcoding species (COI is the convention), and because we found that it sometimes lacked sufficient resolution to distinguish between related species based on short sequence reads. For example, *Oxyjulis californica* and *Halichoeres semicinctus* differ by only a single base substitution in a region of over 380 bp in the center of the 16S amplicon; consequently, on occasions when only short sequences were obtained, these species could not be reliably distinguished. Although we had a significant failure rate in our COI amplifications, analysis of a set of 1,066 of those failures with the 16S rRNA gene did not reveal evidence for COI bias; the composition of species observed in the 16S samples did not differ significantly from the distribution of species identified by COI sequencing.

When compared with other studies of nearby areas, we see some key differences in our results that can highlight the value of ichthyoplankton surveys. For example, though one of the most commonly seen species in our study was the señorita (*O*. *californica*), a previous study of the same MPA that used diver surveys and trawls did not find señorita in their sampling [9]. This can indicate one of the benefits of sampling fish eggs: because señorita typically live near structures (in this case the Scripps Pier), they could be difficult to observe with trawling methods [43]. Similarly, an extensive study of fish entrained in the cooling systems of power plants [3] did not report finding any sanddabs (*Citharichthys* spp.); in contrast, other studies consistently note that sanddabs are the most abundant species in the soft-bottom nearshore community ([9, 10], and the present study). Again, this indicates how different sampling methods can affect the data and suggests that including fish eggs in a monitoring study can provide a more complete picture of the species assemblage.

The probability exposure maps constructed for this study allow us to predict the likelihood of eggs being spawned within or outside the MPA. Though the lack of eggs from offshore species is a good indication that most eggs are spawned in close proximity to our collection site, these maps, based on real-time estimates of surface currents, allowed us to assess the probability that eggs came from distant locales. We selected various collection dates to test in the model based on high diversity, season, and species found. On all these dates, the highest probabilities of exposure were estimated to originate from within or very close to the MPA. On two of the days that we tested with the surface transport model, we found kelp forest species (e.g., *Paralabrax clathratus*) and wanted to test whether those species had come from the kelp forest immediately south of the Scripps Pier. Both days had a northward trend in currents that predicted eggs had advected from just south of the MPA. Due to the increased variability of currents around La Jolla Point, the probability exposure maps in this region need to be interpreted conservatively; however, the presence of kelp species in pier samples and the northward flow of water estimated by the probability maps suggests that the majority of eggs came from within the MPA in both cases. The surface transport model was essential for visualizing the predicted path of eggs and leads us to the conclusion that, in a majority of cases, eggs are most likely to have been spawned within the MPA.

Another important aspect of MPA function concerns the spillover effect. An ideal MPA design protects spawning but allows for advection of larvae out of the MPA to nearby unprotected habitats, thereby enhancing regional recreational and commercial fisheries. The probability exposure maps for larvae show that, although some larvae are likely retained in the MPA, a portion are also advected out of its boundaries. Although we only looked at exposure maps for two specific time periods from this study, we saw a similar pattern of southward movement for both dates, which came from two different seasons in different years. The probability exposure maps were essential in the determination of the general trend in movement experienced by recently hatched fish and suggest that a significant portion of larvae are retained within the MPA while the rest contribute to spillover nearby.

Monitoring studies are extremely useful for both providing an understanding of the fish species assemblage in a study area and for comparison to future studies. Here we found remarkably similar patterns in comparing across two years, with species composition clustering based on month and season for most of the year. The extent to which these years are representative of spawning in this MPA remains to be determined. For the present, they represent a baseline to which future years can be compared. Local spawning indicates that these MPAs serve as potentially important sources of recruits for a significant portion of resident species; continued fish egg monitoring studies can provide early indications of any changes in spawning in the future.

## Supporting Information

S1 Table Percentage of collections in each month from which a given species was identified. For each species with at least 10 eggs identified in this study, we quantified the fraction of collections in which eggs were present in a given month. Red boxes indicate species that were found in at least 90% of collections in a month, orange indicate at least 75%, green at least 50%, blue at least 25%, and purple are greater than 0.
(DOCX)
