# Supplementary material for: Monitoring Spawning Activity in a Southern California Marine Protected Area Using Molecular Identification of Fish Eggs
Source: PLoS One. 2015 Aug 26;10(8):e0134647. doi: 10.1371/journal.pone.0134647 (PMC4550277; doi:10.1371/journal.pone.0134647)
Supplement: S1 Table — For each species with at least 10 eggs identified in this study, we quantified the fraction of collections in which eggs were present in a given month. Red boxes indicate species that were found in at least 90% of collections in a month, orange indicate at least 75%, green at least 50%, blue at least 25%, and purple are greater than 0. (DOCX) [file pone.0134647.s001.docx]

| **Species** | **Year** | **Sep** | **Oct** | **Nov** | **Dec** | **Jan** | **Feb** | **Mar** | **Apr** | **May** | **Jun** | **Jul** | **Aug** |
| --- | --- | --- | --- | --- | --- | --- | --- | --- | --- | --- | --- | --- | --- |
| **Speckled sanddab *(****Citharichthys stigmaeus)* | 2012-2013 | 0.89 | 0.69 | 1.00 | 0.71 | 0.94 | 0.11 | 1.00 | 1.00 | 1.00 | 1.00 | 1.00 | 0.89 |
|  | 2013-2014 | 0.93 | 0.86 | 1.00 | 1.00 | 0.78 | 0.88 | 0.44 | 0.38 | 0.78 | 1.00 | 1.00 | 1.00 |
| **Señorita**  *(Oxyjulis californica)* | 2012-2013 | 0.22 | 0.08 | 0.00 | 0.00 | 0.00 | 0.00 | 0.20 | 0.74 | 1.00 | 1.00 | 1.00 | 0.84 |
|  | 2013-2014 | 0.21 | 0.14 | 0.00 | 0.00 | 0.00 | 0.00 | 0.00 | 0.75 | 0.89 | 1.00 | 1.00 | 1.00 |
| **Pacific sardine** *(Sardinops sagax)* | 2012-2013 | 0.11 | 0.00 | 0.00 | 0.00 | 0.06 | 0.00 | 0.00 | 0.05 | 0.43 | 0.43 | 0.00 | 0.05 |
|  | 2013-2014 | 0.00 | 0.00 | 0.00 | 0.00 | 0.00 | 0.00 | 0.00 | 0.00 | 0.56 | 0.22 | 0.29 | 0.33 |
| **Northern anchovy** *(Engraulis mordax)* | 2012-2013 | 0.00 | 0.00 | 0.00 | 0.14 | 0.38 | 0.22 | 0.60 | 0.26 | 0.29 | 0.00 | 0.05 | 0.00 |
|  | 2013-2014 | 0.07 | 0.00 | 0.00 | 0.14 | 0.33 | 0.50 | 0.11 | 0.00 | 0.11 | 0.00 | 0.00 | 0.22 |
| **California salema** *(Xenistius californiensis)* | 2012-2013 | 0.00 | 0.00 | 0.00 | 0.00 | 0.00 | 0.00 | 0.00 | 0.00 | 0.00 | 0.43 | 0.58 | 0.37 |
|  | 2013-2014 | 0.00 | 0.00 | 0.00 | 0.00 | 0.00 | 0.00 | 0.00 | 0.00 | 0.22 | 0.56 | 0.71 | 0.44 |
| **California corbina** *(Menticirrhus undulatus)* | 2012-2013 | 0.00 | 0.00 | 0.00 | 0.00 | 0.00 | 0.00 | 0.00 | 0.00 | 0.33 | 0.93 | 0.37 | 0.42 |
|  | 2013-2014 | 0.00 | 0.00 | 0.00 | 0.00 | 0.00 | 0.00 | 0.00 | 0.00 | 0.33 | 0.89 | 0.43 | 0.33 |
| **Pacific sanddab** *(Citharichthys sordidus)* | 2012-2013 | 0.11 | 0.23 | 0.75 | 0.43 | 0.13 | 0.00 | 0.20 | 0.47 | 0.90 | 0.93 | 0.58 | 0.58 |
|  | 2013-2014 | 0.29 | 0.43 | 0.71 | 0.14 | 0.11 | 0.38 | 0.00 | 0.00 | 0.11 | 0.56 | 0.29 | 0.44 |
| **Spotfin croaker** *(Roncador stearnsii)* | 2012-2013 | 0.00 | 0.00 | 0.00 | 0.14 | 0.19 | 0.00 | 0.00 | 0.00 | 0.43 | 0.57 | 0.32 | 0.37 |
|  | 2013-2014 | 0.00 | 0.00 | 0.00 | 0.00 | 0.00 | 0.00 | 0.00 | 0.00 | 0.00 | 0.78 | 0.00 | 0.22 |
| **California halibut** *(Paralichthys californicus)* | 2012-2013 | 0.11 | 0.00 | 0.25 | 0.29 | 0.13 | 0.00 | 0.30 | 0.42 | 0.67 | 0.71 | 0.32 | 0.37 |
|  | 2013-2014 | 0.14 | 0.14 | 0.00 | 0.29 | 0.44 | 0.63 | 0.22 | 0.13 | 0.33 | 0.78 | 0.14 | 0.89 |
| **Rock wrasse** *(Halichoeres semicinctus)* | 2012-2013 | 0.33 | 0.00 | 0.00 | 0.00 | 0.00 | 0.00 | 0.00 | 0.00 | 0.29 | 0.86 | 0.42 | 0.63 |
|  | 2013-2014 | 0.29 | 0.14 | 0.00 | 0.00 | 0.00 | 0.00 | 0.00 | 0.00 | 0.11 | 0.89 | 0.43 | 0.56 |
| **Queenfish**  *(Seriphus politus)* | 2012-2013 | 0.00 | 0.00 | 0.00 | 0.00 | 0.00 | 0.00 | 0.00 | 0.00 | 0.29 | 0.36 | 0.37 | 0.16 |
|  | 2013-2014 | 0.00 | 0.00 | 0.00 | 0.00 | 0.00 | 0.00 | 0.00 | 0.00 | 0.00 | 0.78 | 0.14 | 0.33 |
| **Pacific jack mackerel** *(Trachurus symmetricus)* | 2012-2013 | 0.00 | 0.00 | 0.00 | 0.00 | 0.00 | 0.00 | 0.00 | 0.05 | 0.52 | 0.29 | 0.00 | 0.05 |
|  | 2013-2014 | 0.00 | 0.00 | 0.00 | 0.00 | 0.00 | 0.00 | 0.00 | 0.00 | 0.11 | 0.22 | 0.00 | 0.00 |
| **Diamond turbot** *(Hypsopsetta guttulata)* | 2012-2013 | 0.33 | 0.46 | 0.25 | 0.00 | 0.44 | 0.33 | 0.40 | 0.26 | 0.19 | 0.07 | 0.00 | 0.21 |
|  | 2013-2014 | 0.14 | 0.57 | 0.14 | 0.00 | 0.33 | 0.38 | 0.22 | 0.00 | 0.00 | 0.11 | 0.00 | 0.11 |
| **Kelp bass**  *(Paralabrax clathratus)* | 2012-2013 | 0.00 | 0.00 | 0.00 | 0.00 | 0.00 | 0.00 | 0.00 | 0.00 | 0.14 | 0.29 | 0.21 | 0.42 |
|  | 2013-2014 | 0.07 | 0.00 | 0.00 | 0.00 | 0.00 | 0.00 | 0.00 | 0.00 | 0.33 | 0.67 | 0.57 | 0.33 |
| **Sheephead** *(Semicossyphus pulcher)* | 2012-2013 | 0.11 | 0.08 | 0.00 | 0.00 | 0.00 | 0.00 | 0.00 | 0.00 | 0.19 | 0.29 | 0.26 | 0.53 |
|  | 2013-2014 | 0.14 | 0.29 | 0.00 | 0.00 | 0.00 | 0.00 | 0.00 | 0.00 | 0.11 | 0.56 | 0.14 | 0.33 |
| **Longfin sanddab** *(Citharichthys xanthostigma)* | 2012-2013 | 0.00 | 0.08 | 0.00 | 0.00 | 0.06 | 0.00 | 0.10 | 0.42 | 0.24 | 0.14 | 0.26 | 0.11 |
|  | 2013-2014 | 0.00 | 0.14 | 0.14 | 0.29 | 0.00 | 0.25 | 0.00 | 0.00 | 0.00 | 0.11 | 0.00 | 0.22 |
| **Black croaker** *(Cheilotrema saturnum)* | 2012-2013 | 0.00 | 0.00 | 0.00 | 0.00 | 0.00 | 0.00 | 0.00 | 0.16 | 0.33 | 0.07 | 0.11 | 0.05 |
|  | 2013-2014 | 0.00 | 0.00 | 0.00 | 0.00 | 0.00 | 0.00 | 0.00 | 0.00 | 0.44 | 0.33 | 0.14 | 0.00 |
| **Chub mackerel** *(Scomber japonicus)* | 2012-2013 | 0.00 | 0.00 | 0.00 | 0.00 | 0.00 | 0.00 | 0.00 | 0.00 | 0.19 | 0.21 | 0.11 | 0.00 |
|  | 2013-2014 | 0.00 | 0.00 | 0.00 | 0.00 | 0.00 | 0.00 | 0.00 | 0.00 | 0.00 | 0.44 | 0.00 | 0.00 |
| **White croaker** *(Genyonemus lineatus)* | 2012-2013 | 0.00 | 0.00 | 0.00 | 0.00 | 0.19 | 0.00 | 0.00 | 0.05 | 0.05 | 0.00 | 0.16 | 0.00 |
|  | 2013-2014 | 0.00 | 0.00 | 0.00 | 0.14 | 0.00 | 0.13 | 0.11 | 0.00 | 0.00 | 0.33 | 0.00 | 0.00 |
| **Xantic sargo** *(Anisotremus davidsonii)* | 2012-2013 | 0.00 | 0.00 | 0.00 | 0.00 | 0.00 | 0.00 | 0.00 | 0.00 | 0.05 | 0.07 | 0.00 | 0.05 |
|  | 2013-2014 | 0.00 | 0.00 | 0.00 | 0.00 | 0.00 | 0.00 | 0.00 | 0.00 | 0.00 | 0.22 | 0.29 | 0.22 |
| **Pacific pompano** *(Peprilus simillimus)* | 2012-2013 | 0.00 | 0.00 | 0.00 | 0.14 | 0.13 | 0.00 | 0.10 | 0.05 | 0.10 | 0.07 | 0.00 | 0.00 |
|  | 2013-2014 | 0.00 | 0.00 | 0.29 | 0.29 | 0.11 | 0.25 | 0.00 | 0.00 | 0.11 | 0.00 | 0.00 | 0.00 |
| **Shortfin weakfish** *(Cynoscion parvipinnis)* | 2012-2013 | 0.00 | 0.00 | 0.00 | 0.00 | 0.00 | 0.00 | 0.00 | 0.00 | 0.10 | 0.43 | 0.11 | 0.00 |
|  | 2013-2014 | 0.00 | 0.00 | 0.00 | 0.00 | 0.00 | 0.00 | 0.00 | 0.00 | 0.11 | 0.11 | 0.00 | 0.00 |
| **Spotted cusk-eel** *(Chilara taylori)* | 2012-2013 | 0.11 | 0.00 | 0.00 | 0.00 | 0.00 | 0.00 | 0.10 | 0.00 | 0.10 | 0.07 | 0.11 | 0.16 |
|  | 2013-2014 | 0.00 | 0.00 | 0.14 | 0.00 | 0.00 | 0.00 | 0.00 | 0.00 | 0.00 | 0.33 | 0.00 | 0.00 |
| **White seabass** *(Atractoscion nobilis)* | 2012-2013 | 0.00 | 0.00 | 0.00 | 0.00 | 0.00 | 0.00 | 0.00 | 0.00 | 0.05 | 0.00 | 0.00 | 0.00 |
|  | 2013-2014 | 0.00 | 0.00 | 0.00 | 0.00 | 0.00 | 0.00 | 0.00 | 0.13 | 0.22 | 0.22 | 0.14 | 0.00 |
| **California tonguefish** *(Symphurus atricaudus)* | 2012-2013 | 0.00 | 0.15 | 0.00 | 0.00 | 0.00 | 0.00 | 0.00 | 0.00 | 0.00 | 0.07 | 0.05 | 0.11 |
|  | 2013-2014 | 0.00 | 0.00 | 0.14 | 0.00 | 0.00 | 0.00 | 0.00 | 0.00 | 0.00 | 0.00 | 0.00 | 0.33 |
| **Barred sand bass** *(Paralabrax nebulifer)* | 2012-2013 | 0.00 | 0.00 | 0.00 | 0.00 | 0.00 | 0.00 | 0.00 | 0.00 | 0.00 | 0.07 | 0.11 | 0.21 |
|  | 2013-2014 | 0.00 | 0.00 | 0.00 | 0.00 | 0.00 | 0.00 | 0.00 | 0.00 | 0.00 | 0.11 | 0.00 | 0.22 |
| **Fantail sole**  *(Xystreurys liolepis)* | 2012-2013 | 0.00 | 0.23 | 0.00 | 0.00 | 0.00 | 0.00 | 0.00 | 0.00 | 0.00 | 0.00 | 0.00 | 0.00 |
|  | 2013-2014 | 0.07 | 0.00 | 0.00 | 0.00 | 0.00 | 0.00 | 0.00 | 0.00 | 0.00 | 0.00 | 0.00 | 0.11 |
| **Yellowfin croaker** *(Umbrina roncador)* | 2012-2013 | 0.00 | 0.00 | 0.00 | 0.00 | 0.00 | 0.00 | 0.00 | 0.00 | 0.00 | 0.21 | 0.05 | 0.00 |
|  | 2013-2014 | 0.00 | 0.00 | 0.00 | 0.00 | 0.00 | 0.00 | 0.00 | 0.00 | 0.00 | 0.11 | 0.00 | 0.00 |
